# Symptoms of Mental Health Problems: Children’s and Adolescents’ Understandings and Implications for Gender Differences in Help Seeking

**DOI:** 10.1111/j.1099-0860.2011.00406.x

**Published:** 2013-05

**Authors:** Alice MacLean, Kate Hunt, Helen Sweeting

**Affiliations:** MRC Social and Public Health Sciences Unit, University of GlasgowGlasgow, G12 8RZ, UK

**Keywords:** adolescence, childhood, health and well-being, mental health, help seeking

## Abstract

Amidst concerns that young people’s mental health is deteriorating, it is important to explore their understandings of symptoms of mental health problems and beliefs around help seeking. Drawing on focus group data from Scottish school pupils, we demonstrate how they understood symptoms of mental health problems and how their characterisations of these symptoms as ‘rare’ and ‘weird’ informed participants’ perceptions that peers, teachers and parents would respond to disclosure in stigmatising ways. Consequently, participants suggested that they would delay or avoid disclosing symptoms of mental health problems. We highlight subtle gender and age differences and outline implications for policy and practice.

## Introduction

There is growing concern that the mental health of young people has deteriorated over recent decades (Rutter and Smith, 1995; Sweeting and others 2010). Low levels of help seeking and mental health service use in adolescence and early adulthood (Potts and others, 2001; Rickwood and Braithwaite, 1994), combined with high prevalence of mental health symptoms and psychological disorders (Green and others, 2005; Williams and Pow, 2007), suggest a need for targeted mental health promotion. If early intervention or prevention of serious mental health problems, such as depression, is to be achieved, it is important to understand the ways in which symptoms of mental health problems, such as feeling sad, anxious or irritable, and the consequences of seeking help for them, are understood by children and adolescents.

It is widely reported that during early-mid adolescence there are changes in the gender distribution of psychological distress (Torsheim and others, 2006; Wade and others, 2002): generally high rates of self-reported symptoms of mental health problems in early adolescence increase more steeply with age for girls, resulting in a marked female excess by mid adolescence. For example, a survey of 2063 school pupils found that 33 per cent male pupils and 37 per cent female pupils reported ‘feeling sad, unhappy or low’ in the past month at age 11, whereas by age 15 the rates were 32 per cent male pupils and 56 per cent female pupils (Sweeting and West, 2003). Although little attention has been paid to explaining this pattern, one plausible explanation is that girls are more willing to report symptoms of mental health problems than boys. However, few studies have investigated age or gender differences in understandings of symptoms of mental health problems and their impact upon willingness to report and seek help.

Before outlining the aims of this article, we review what is known about understandings of mental health and illness, perceptions of the consequences of their disclosure and whether or not each of these varies according to age and gender, during the transition from late childhood to early adulthood.

### Understandings of mental health and illness: do they vary according to age and gender?

Studies which have explored children’s (Roose and John, 2003), adolescents’ (Armstrong and others, 2000; Johansson and others, 2007; Williams and Pow, 2007) and young adults’ (Biddle and others, 2006) understandings of health suggest participants distinguish between physical and mental health. In contrast to physical illness, which is perceived as adverse visible changes to a person’s body (Johansson and others, 2007), mental health problems are seen as emotional experiences encompassing how you feel and think (Landstedt and others, 2009; Secker and others, 1999). However, some age and gender differences have been highlighted. Johansson and others (2007) found that 13-year-olds had more difficulty than 16-year-olds in understanding the concept of ‘mental health’. In relation to gender, evidence suggests that girls have higher levels of knowledge (Williams and Pow, 2007), mental health literacy (Burns and Rapee, 2006) and are more expressive in talking about poor mental health (Johansson and others, 2007).

Across age and gender, the concept of normality is central; being mentally healthy is equated with being ‘normal’ or *not*‘different’ (Armstrong and others, 2000). Emotional states which children and adolescents commonly experience (such as anger or sadness) are understood as ‘normal’ whereas behaviours they have difficulty identifying with (such as people talking to themselves), or which are unusual for the person experiencing them (such as crying without justification), are understood as mental illness (Roose and John, 2003; Secker and others, 1999). Biddle and others (2007) found that 16- to 24-year-olds with mental health problems also draw on notions of normality when constructing their understandings of mental distress. They conceptualised ‘normal’ distress, ranging from minor stress to severe depression, as ‘universally and inevitably experienced throughout life in response to common life events and stresses’ (p. 989). At the other end of the spectrum, ‘real’ distress or mental illness, including conditions such as schizophrenia, was abnormal and rare.

### Beliefs about the consequences of disclosing mental illness: do they vary according to age and gender?

Across age and gender, notions of normality are also central to beliefs about the consequences of help seeking for mental health problems. Children and young people fear help seeking will lead to them being seen, and treated, as ‘different’ and a ‘problem’ by peers, teachers and family (Biddle and others, 2007; Roose and John, 2003; Spratt and others, 2010). American 13-year-olds believed that if someone their age was using mental health services they would be made fun of, labelled weird and treated as an outcast by peers (Chandra and Minkovitz, 2007). Among 60 American adolescent mental health service users, 45 per cent reported, during semi-structured interviews, that they had experienced some peer stigma (treated differently compared to others or to how they were treated before using services) while, 18 per cent described experiencing substantial stigma through peer alienation (Moses, 2010). Earlier analyses of data from participants in the present study suggested that adolescents’ general beliefs that physical illness is more common and therefore ‘more normal’ than mental health problems, lead them to expect disclosures of mental health problems will be met by more stigmatising responses than disclosures of physical symptoms (MacLean and others, 2010).

Past studies suggest that the context in which mental health problems are disclosed affects the perceived stigma: responses are thought to be most punitive in school and peer (rather than private family) contexts, especially for boys (Johansson and others, 2007; Moses, 2010). Underlying this belief are stereotypical gender expectations that it is ‘normal’, and perhaps expected, for girls to be ‘emotional’ whereas it is not ‘manly’ for boys to have, or seek help for, symptoms of mental health problems (Roose and John, 2003; Timlin-Scalera and others, 2003). Not only are boys believed to suffer more stigmatising responses to mental health problems, but they have also been found to hold more negative and stigmatising attitudes to people experiencing these (Williams and Pow, 2007).

To avoid stigmatising responses, boys and girls of all ages from late childhood to early adulthood suggest that they would internalise symptoms of mental health problems, and try to cope with them independently (Armstrong and others, 2000; Johansson and others, 2007; MacLean and others, 2010) and conceal mental health service use (Moses, 2010). In a survey of 496 fifteen- to sixteen-year-old Scottish school pupils, 44 per cent indicated that they would not want other people to know if they had a mental health problem (Williams and Pow, 2007). Biddle and others (2007) demonstrate the lengths to which young adults with mental health problems go to convince themselves that their symptoms are ‘normal’ to delay help seeking; indeed they see the act of going for help as triggering an ‘irreversible status passage’ through which they lose their ability to stay ‘normal’ and become known as someone with ‘real’ mental distress. Although studies suggest that school-aged boys and girls would attempt to conceal psychological distress, it appears that girls are more likely to eventually seek help (Santor and others, 2007).

Existing research is based around children’s and young people’s understandings of mental health and illness as general concepts. However, little is known about how they understand *specific* symptoms of mental health problems, such as feeling sad, anxious or irritable, which may represent initial manifestations of more serious mental health problems, such as depression. Also it is not known whether or not such understandings impact upon help seeking. Our review of existing literature revealed that only one study (Johansson and others, 2007) systematically compared children’s and young people’s understandings of mental health across gender and age; and only one (Roose and John, 2003) focused on those younger than 13. If we are to understand more about why the gender distribution of psychological distress changes during early-mid adolescence it is important to systematically compare children’s and adolescents’ understandings of symptoms of mental health problems and their perceptions of help seeking by gender and age.

In this article, we use data from task-centred focus groups to:

- explore 10-, 13- and 15-year-olds’ understandings of symptoms of mental health problems;
- investigate how these understandings impact upon participants’ beliefs around whether or not they would seek help; and
- systematically examine any age or gender differences in understandings of symptoms of mental health problems and perceptions of help seeking which may help to explain the emerging female gender excess in self-reported psychological morbidity during early-mid adolescence.

## Method

### Participants

Participants were recruited from one secondary and its main associated primary school in a town within a predominantly rural area (MacLean, 2006). Both schools have a large, mixed catchment, including pupils from working class, farming and middle class backgrounds. As all participants were White Scottish, reflecting lack of ethnic diversity in the area, we do not consider the impact of ethnicity on understandings of symptoms of mental health problems and perceptions of help seeking among children and adolescents. Due to time constraints when conducting research in schools, details of parental occupation or other indicators of participants’ socioeconomic status were not gathered.

### Ethics

Teachers were asked to distribute information packs, including parental opt-out consent forms, to as wide a range of pupils as possible. Only one parental withdrawal was received. In accordance with the protocol approved by Glasgow University ethics committee, relevant Education Authorities and head teachers of the participating schools, we relied on the method which the schools themselves most frequently use to communicate with parents, i.e. delivery of study information and consent forms via the child, to maintain confidentiality of home addresses. It is possible that a parent may not have had the opportunity to opt-out their child’s participation if the child did not pass on the pack. Informed consent was obtained from all participants. They were given leaflets which explained the study and included the researcher’s contact details should they wish further information. Before focus groups began, it was explained to participants that they were under no obligation to take part and could withdraw at any time. They were then asked to sign a consent form to indicate that they had read the information leaflet, been given the opportunity to ask questions, and agreed to take part in the research.

Consideration was also given to ethical issues which may arise from pupils participating in discussions about symptoms in the presence of peers and steps were taken to minimise any negative impact. Focus group questions and tasks were tailored so that pupils did not have to discuss their *own* experiences of symptoms. At the beginning of each group, the importance of discretion was emphasised and pupils were asked to treat the discussion as confidential. Protocols were developed for dealing with any evident distress among pupils during the course of the research. Information leaflets distributed at the beginning of each focus group explained that, should anyone disclose that they may be at risk of serious harm, the researcher would need to discuss with that individual the best way to inform appropriate adults or agencies to address the situation. All pupils were also provided information on services they could access for confidential advice (e.g. ChildLine and relevant websites).

### Procedures

Focus groups were conducted within school time and on school premises. All were single sex with pupils drawn from the same year group. Focus groups can have ‘ethnographic potential’ in leading to the enactment or mimicking of the cultural processes which participants claim to be normative practices (Hyde and others, 2005). They were used in this study to reveal cultural norms surrounding boys’ and girls’ understandings of a range of symptoms and their perceptions of help seeking in various social contexts. Whereas acknowledging that the presence of friends in focus groups can have negative as well as positive effects on interactions (Michell, 1999), we attempted to recruit friendship groups to create an environment in which participants should feel safer about sharing their thoughts, even if their views diverged from others in the group (Morgan, 1997). Within the primary school, teachers selected friendship groups from all those wanting to take part and the timing was flexible, although groups generally lasted about an hour. Greater limitations were imposed by the curriculum and other factors in the secondary school. Here, groups were selected by teachers from Social Education classes, so that close friendships within groups were more by chance than design, and the school timetable constrained discussions to a maximum of 40 min.

Table 1 provides details of the study sample and fieldwork conducted. In total, 90 pupils from three year groups participated in 25 focus groups. Henceforth the groups are termed 10-, 13- and 15-year-olds. All discussions were audio-taped and transcribed; field-notes were written during and after each group.

**Table 1:** Details of study sample and fieldwork conducted

	Primary six pupils (average age 10 years 10 months)		Secondary two pupils (average age 13 years 1 month)		Secondary four pupils (average age 15 years 1 month)		
				
	Girls	Boys	Girls	Boys	Girls	Boys	Total
Focus groups	5	5	3	4	4	4	25
Participants	21	21	10	13	13	12	90

### Materials

Researchers have debated use of specifically designed research methods for children (Harden and others, 2000; Punch, 2002). Our focus groups were designed to be task-centred, to render symptom reporting less abstract, and aid comparison across gender and age groups. The topic guide included three exercises, two of which generated data presented in this article. The first featured a set of 10 cards, each with a *‘physical’* (‘stomach ache or feeling sick’; ‘headache’; ‘aching back, legs or arms’; ‘cold or flu’; ‘asthma or wheezy chest’) or a *‘psychological’* symptom (‘nervous, worried or anxious’; ‘sad, unhappy or low’; ‘difficulty getting to sleep’; ‘irritable or bad tempered’; ‘dizzy or faint’) written on it. We acknowledge difficulties in drawing clear distinctions between ‘*physical*’ and ‘*psychological*’ symptoms (Macintyre and others, 1996). Participants were asked: which symptoms they thought that they, and their opposite-sex peers, would be most, and least, likely to seek help for and why; and which symptoms either boys or girls were more likely to get and why. The second task involved a series of four vignettes in which a boy (‘Steven’) and a girl (‘Sarah’), of the same age as participants, were described as suffering first a stomach ache and then feeling like she/he was ‘going to cry all the time’ in three different social contexts (in class, out with friends and at home, presented separately in that order). Participants discussed what the same-gender character might do about each symptom before discussing the opposite-gender character.

### Analyses

Descriptive analysis identified important themes across the data and entailed systematic comparison across gender and age groups. The software program NVivo was used to code themes relating to interpretations and experience of symptoms, gender and age-related expectations and physical and social consequences of symptom reporting. Coding was broad to avoid removing data from context and to allow for multiple interpretations of ambiguous sections. Initially, all authors read transcripts and discussed broad themes and codes. These were subsequently refined and organised using a system based on the ‘Framework’ approach (Ritchie and Lewis, 2003), enabling identification and description of patterns, associations and irregularities within the data.

## Results

### How are common symptoms understood by children and teenagers?

Participants’ understandings of the 10 symptoms were complex and multi-dimensional (see MacLean, 2006 for a full exploration), but ‘*physical*’ and ‘*psychological*’ symptoms were clearly distinguished as oppositional in various ways. Participants grouped together certain symptoms (‘cold or flu’; ‘headache’; ‘stomach ache or feeling sick’; ‘asthma or wheezy chest’; ‘dizzy or faint’ and ‘aching back, legs or arms’), characterising most as ‘painful’, ‘physical’, ‘ubiquitous’, ‘normal’, ‘treatable’, ‘real illness’ and ‘involuntary’. With the exception of ‘dizzy or faint’, these were all ‘*physical*’ symptoms. In contrast, most ‘*psychological*’ symptoms (‘feeling irritable or bad tempered’; ‘sad, unhappy or low’; ‘nervous, worried or anxious’; ‘difficulty getting to sleep’ and the vignette symptom ‘feeling like crying all the time’) were characterised as ‘painless’, ‘emotional’, ‘rare’, ‘weird’, ‘taboo’, ‘untreatable’, ‘not illness’ and ‘voluntary’.

**Figure 1:**
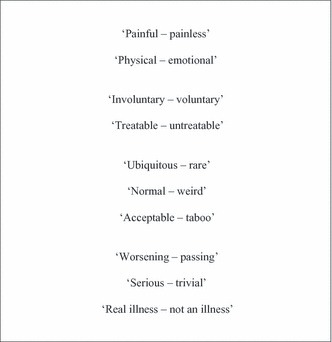
Making Sense of Symptoms: ‘Conceptual Continua’ Drawn on by Participants to Construct Their Understandings of Symptoms.

Our analyses suggested a number of what we have termed ‘conceptual continua’ to describe how participants constructed their understandings of symptoms (Figure 1). In the following sections we explore participants’ understandings of ‘*psychological*’ symptoms in more detail, paying attention to the ways in which these informed discussions about whether or not they would seek help for symptoms and, in turn, perceptions of how others might respond. It is important to note that our analyses uncovered surprisingly few significant gender or age differences across accounts. The following two sections outline those understandings which did not vary by gender or age (*‘psychological’* symptoms as ‘painless’ and ‘emotional’; ‘voluntary’ and ‘untreatable’). Understandings which varied by gender or age (*‘psychological’* symptoms as ‘rare’, ‘weird’ and ‘taboo’; more likely to be ‘passing’, ‘trivial’ and ‘not an illness’ for boys, but ‘worsening’, ‘serious’ and ‘real illness’ for girls) are dealt with in the third and fourth sections.

### ‘Psychological’ symptoms as ‘painless’ and ‘emotional’

Most participants referred to ‘irritable or bad tempered’, ‘sad, unhappy or low’, ‘nervous, worried or anxious’ and ‘difficulty getting to sleep’ as symptoms that ‘you wouldn’t really notice’ (James, 15) and ‘don’t make you feel sore as much’ (Leigha, 10). Participants also contrasted the vignette symptoms in this way. For instance, Keith (13) described ‘feeling like crying all the time’ as an ‘expressive feeling’, but stomach ache as a ‘sore, painful thing’. ‘*Psychological*’ symptoms were also described as ‘emotional’ by referring to ‘irritable or bad tempered’; ‘sad, unhappy or low’; and ‘nervous, worried or anxious’ as ‘feelings’ (Ellie, 10) or ‘emotional sort of [symptoms]’ (Amy, 1*5*).

Based on these understandings, participants suggested that they would be more likely to seek help for ‘*physical*’ than ‘*psychological*’ symptoms to get rid of the pain they caused.

### ‘Psychological’ symptoms as ‘voluntary’ and ‘untreatable’

Symptoms such as ‘irritable or bad tempered’, ‘sad, unhappy or low’ and ‘nervous, worried or anxious’, were understood as those that ‘you have the choice of feeling’ (Ellie, 10). Similar sentiments were echoed as Liam (10) claimed that ‘you can’t help’ stomach ache but ‘you can kind of help crying if you want to’ and Pamela (15) that boys would report symptoms such as ‘cold or flu’ and ‘aching back, legs or arms’ because they ‘[are] not anything [that’s] their fault’. Implicit in these suggestions was the idea that it is a failing or sign of weakness to suffer from ‘voluntary’ symptoms, because, being supposedly controllable, they should not be ‘allowed’ to develop.

Most participants suggested that ‘feeling like crying all the time’ is ‘untreatable’. For example:

James: […] if it’s a stomach ache, you know, just …

Kevin: Take a Paracetamol.

James: Get to bed early.

Kevin: See what it’s like in the morning.

**Interviewer: Mhmm.**

James: Whereas depression [‘feeling like crying all the time’] is, it’s not like you can just take a … Paracetamol and it’ll go away.

(15-year-old boys)

However, a few suggested that ‘feeling like crying all the time’ could be treated. For example, Becky (15) claimed:

[…] if you went to your doctor and you’re like ‘Oh, I’m sad and unhappy quite a lot’, they’ll end up just putting you on anti-depressants or something, even if you aren’t depressed, cos that’s the only thing they can do.

(15-year-old girls)

When speculating about the consequences of reporting ‘feeling like crying all the time’, participants’ perceptions were informed by beliefs that this is ‘untreatable’:

**Interviewer: And what about how other people might react to that one** [**‘feeling like crying all the time**]**?**

Lewis: Might treat you differently.

**Interviewer: Mhmm. And what … how would people react to it if you were ill with a stomach ache?**

Lewis: Just give you some tablets or send you to bed or somethin’… until it goes away.

(13-year-old boys)

Lewis suggested that a stomach ache can be treated *directly* and *in isolation* from the sufferer more broadly, whereas in the case of ‘feeling like crying all the time’, it would be the *person experiencing the symptom* who would be treated differently. This implies participants assessed ‘stomach ache’ according to its potential bodily effects but ‘feeling like crying all the time’ in terms of the potential consequences of its disclosure on their social identities. The possibility that seeking help for this symptom would lead to being treated ‘differently’ was alluded to as a deterrent to disclosure by both boys and girls.

### ‘Psychological’ symptoms as ‘rare’, ‘weird’ and ‘taboo’

Participants commonly differentiated between symptoms which ‘happen to everybody’ (Pamela, 15), such as ‘colds or flu’, ‘headache’ and ‘stomach ache or feeling sick’ and those which ‘people don’t normally get’ (Sandra, 10), such as ‘difficulty getting to sleep’ and ‘sad, unhappy or low’, for example:

Nick: Everyone gets a stomach ache but not everyone might — not everyone will get depressed.

(13-year-old boys)

Some also described ‘feeling like crying all the time’ as ‘weird’ in comparison to stomach ache. For instance, Rose (15) claimed that ‘stomach ache’s a bit more of a normal thing to get’ whereas crying in class ‘would seem a bit weird’.

Understandings of ‘feeling like crying all the time’ as ‘rare’ and ‘weird’ influenced perceptions of peers’, teachers’ and parents’ responses to its disclosure. This example is typical:

Calum: Going home with a sore stomach’s not really a big deal, but crying in class would be pretty …

Liam: That’s pretty embarrassing.

[…]

Calum: … everyone would just talk behind your back about you … if you cried. If you’re sick, people are sick every day in our class and they have to go home […] but no-one really gives a big deal about that.

(10-year-old boys)

Gender- and age-related expectations were apparent in relation to whether a symptom was conceptualised as ‘acceptable’ or ‘taboo’. Boys, across all ages, were adamant they would conceal ‘feeling like crying all the time’, or disguise it as a symptom with masculine connotations (like a sports injury), suggesting they viewed it as a ‘feminised’, ‘taboo’ symptom, threatening their masculine identities. For example:

Kenny: … you shouldn’t really show your feelings at school […] other people don’t so you don’t.

Adam: Especially when you’re like a boy that’s thirteen …

(13-year-old boys)

Both boys’ and girls’ accounts suggested that ‘feeling irritable or bad tempered’ has masculine connotations, and was therefore more ‘acceptable’ for boys but more ‘taboo’ for girls. By claiming irritability is ‘the only [symptom boys] would actually maybe tell or show’, Craig (10) implied that this ‘*psychological*’ symptom is acceptably ‘masculine’. In contrast, girls said they would conceal this symptom:

Sheena: I always think boys as more bad tempered than girls most of the time. But maybe they just do it in more anger, whereas girls hide it or something like that […] they don’t want people to know what they’re feeling, or …

Pamela: I think girls get upset and boys get angry.

(15-year-old girls)

All participants suggested that ‘difficulty getting to sleep’ and crying in class were ‘taboo’ because they were abnormal for their age. Gareth (15) asserted that ‘things like difficulty getting to sleep are things younger children generally have problems with’ and it was generally felt that crying in public without warning or justification would be seen as ‘babyish’ (Tara, 13) and ‘silly’ (Kirsten, 9). Therefore, participants claimed that they would not tell anyone if they had difficulties getting to sleep and would avoid crying in public, especially peer contexts, at all costs.

### ‘Psychological’ symptoms as more likely to be ‘passing’, ‘trivial’ and ‘not an illness’ for boys, but ‘worsening’, ‘serious’ and ‘real illness’ for girls

A discernible, yet subtle, gender difference in relation to understandings of ‘*psychological*’ symptoms was that most boys suggested they are ‘passing’, whereas girls were more likely to conceptualise them as ‘worsening’. For example, Carla (15) suggested that ‘sad, unhappy or low’ and ‘nervous, worried or anxious’ could ‘turn into something worse’, but these were referred to by boys as symptoms ‘that will go away’ (Adam, 13). Only a minority of girls suggested that ‘*psychological*’ symptoms, such as ‘feeling sad, unhappy or low’, ‘might just pass over’ (Rhona, 10).

Girls conceptualised most ‘*psychological*’ symptoms as ‘kind of like more serious’ (Anna, 10) and ‘quite important’ (Vicky, 13). However, ‘feeling irritable or bad tempered’ and ‘difficulty getting to sleep’ were characterised by some as ‘not that serious’ (Sheena, 15). Among the boys, only ‘difficulty getting to sleep’ was understood as being ‘more, like, serious’ (Gregor, 13) and they more commonly conceptualised ‘*psychological*’ symptoms as ‘trivial’.

Participants also contrasted symptoms according to whether or not they viewed them as representing ‘real illness’. This was most common among 13- and 15-year-old boys, although a minority of girls also talked in these terms. ‘Headache’; ‘stomach ache or feeling sick’; ‘asthma or wheezy chest’; ‘dizzy or faint’; ‘aching back, legs or arms’; and ‘cold or flu’ were described by girls and boys as ‘actual illnesses’ (Rose, 15), ‘something wrong with you’ (Matthew, 15) and symptoms that ‘you can get sick with’ (Jack, 10), whereas ‘sad, unhappy or low’; ‘irritable or bad tempered’; and ‘nervous worried or anxious’ were conceptualised, more strongly by boys than girls, as ‘not feeling ill’ (Joe, 15) and symptoms that ‘you can’t really get […] sick with’ (Jack, 10).

Boys were more likely to describe ‘feeling like crying all the time’ as ‘not an illness’. For example:

Craig: [If Steven felt like crying in class] I don’t think he would want to go home, though, because then everyone would just say …

Robert: Yeah, cos there’s nothing wrong with you.

Craig: There’s nothing wrong with you. There’s … just … like, sad.

(10-year-old boys)

In contrast, girls did not describe ‘feeling like crying all the time’ in terms of whether or not it represented ‘real illness’. Paula (15) was the only girl to claim that this was ‘maybe not an illness or anything’.

## Discussion

This study is the first to explore systematically how understandings of a range of symptoms of mental health problems vary by age and gender in late childhood and early-mid adolescence, and to look at how these understandings impact upon beliefs about help seeking. Such differences may contribute to our understanding of the changing gender distribution of psychological morbidity during early-mid adolescence. Echoing previous research demonstrating young people’s tendencies to distinguish between mental and physical aspects of health (Johansson and others, 2007; Landstedt and others, 2009; Roose and John, 2003), participants conceptualised physical and psychological symptoms as different and distinct, not only in how they are experienced and how they would deal with them, but also in how they expected others to react to them.

The results suggested that understandings of symptoms of mental health problems are largely consistent across age and gender. Boys and girls across all age groups said they would be less likely to seek help for symptoms of mental health problems than those indicative of physical illness, consistent with previous reports of children’s and young people’s reluctance to disclose mental health problems (Armstrong and others, 2000; Biddle and others, 2007; Chandra and Minkovitz, 2007). The main deterrent was participants’ understandings of symptoms of mental health problems as being rare and consequent expectations of stigmatising responses from peers, parents and teachers. Their accounts also implied that because these symptoms are particularly unexpected of boys they serve to threaten valued masculine identities at a life stage when consciousness of the need to ‘do gender’‘well’ (Williams, 2000) is highly salient and age-appropriate gender identities are being actively (re)formed. These findings reflect those of Johansson and others (2007) that due to stereotypical expectations, boys feel under more pressure than girls to conceal mental health problems. However, as we have reported previously, we found only very subtle differences between boys’ and girls’ accounts of the consequences of help seeking. Girls also expressed worries about being seen as weak or different but, compared to boys, there was less of a sense that their entire identity would be questioned as a result (MacLean and others, 2010). Any gender or age differences in participants’ understandings of symptoms, and how these influence decisions about help seeking, do not seem large enough to explain the marked female gender excess in psychological distress which emerges during early-mid adolescence.

### Limitations

It is important to note a number of limitations to this study. First, it is possible that procedures used to obtain parental consent, which relied on pupils to deliver study information and opt-out consent forms, may have meant that some parents were denied the opportunity to veto their child’s participation. However, these procedures were approved by a Glasgow University ethics committee, relevant Education Authorities and head teachers. Second, the lack of indicators of socioeconomic status prevents an exploration of the extent to which the findings of this study are class specific. Third, the primary school focus groups were composed of friendship groups selected by teachers. As teachers may not always be the best judge of pupils’ friendships, we acknowledge this as a potential source of bias.

### Implications for policy and practice

Previous research suggests children and young people should be given ‘opportunities for individual learning about mental illness in ways which take [their] own perspectives into account’ (Secker and others, 1999: 738). Participants’ accounts suggested that perceptions of stigma associated with symptoms of mental health problems acted as a significant barrier to help seeking. Implications for health promotion are that campaigns to tackle stigma surrounding mental health problems, such as ‘See Me Scotland’, would benefit from taking boys’ and girls’ understandings into account. Specifically, this study highlights a need to address the misconception that symptoms of mental health problems are rare by educating children and young people about their prevalence, causes, effects and treatments. The findings suggest willingness to seek help for mental health problems may improve if symptoms were understood as ubiquitous or normal, similar to most physical symptoms. Boys’ help seeking could be improved by addressing perceptions that only girls suffer symptoms of mental health problems, although as this conception is deeply culturally entrenched it may be difficult to challenge. However, given evidence that young adults with mental health problems often delay help seeking by convincing themselves that their symptoms are common (Biddle and others, 2007), it is important that helping children and young people to perceive such symptoms as normal does not mean they dismiss them. Instead, as Spratt and others (2010):491) suggest, health promotion services and campaigns should aim to encourage ‘children’s competence to know when they need help and their right to look for it’.

The fact that children as young as 10 distinguish physical and psychological symptoms on grounds of presumed rarity and weirdness, suggests that health promotion around mental illness should begin in primary school. This study also highlights the importance to boys and girls of peers’, parents’ and teachers’ responses to disclosure of mental health symptoms and the ways in which expectations of stigmatising responses may serve to delay or prevent help seeking. Thus, health promotion programmes should be aimed at both children and young people and those who care for and work with them.

## References

[b1] Armstrong C, Hill M, Secker J (2000). Young people’s perceptions of mental health. Children & Society.

[b2] Biddle L, Donovan JL, Gunnell D, Sharp D (2006). Young adults’ perceptions of GPs as a help source for mental distress: a qualitative study. British Journal of General Practice.

[b3] Biddle L, Donovan J, Sharp D, Gunnell D (2007). Explaining non-help seeking amongst young adults with mental distress: a dynamic interpretive model of illness behaviour. Sociology of Health & Illness.

[b4] Burns JR, Rapee RM (2006). Adolescent mental health literacy: young people’s knowledge of depression and help seeking. Journal of Adolescence.

[b5] Chandra A, Minkovitz CS (2007). Factors that influence mental health stigma among 8th grade adolescents. Journal of Youth & Adolescence.

[b6] Green H, McGinnity A, Meltzer H, Ford T, Goodman R (2005). Mental Health of Children and Young People in Great Britain, 2004.

[b7] Harden J, Scott S, Backett-Milburn K, Jackon S (2000). Can’t talk, won’t talk? Methodological issues in researching children. Sociological Research Online.

[b8] Hyde A, Howlett E, Brady D, Drennan J (2005). The focus group method: insights from focus group interviews on sexual health with adolescents. Social Science and Medicine.

[b9] Johansson A, Brunnberg E, Eriksson C (2007). Adolescent girls’ and boys’ perceptions of mental health. Journal of Youth Studies.

[b10] Landstedt E, Asplund K, Gadin KG (2009). Understanding adolescent mental health: the influence of social processes, doing gender and gendered power relations. Sociology of Health & Illness.

[b11] Macintyre S, Hunt K, Sweeting H (1996). Gender differences in health: are things really as simple as they seem?. Social Science & Medicine.

[b12] MacLean A (2006). Rules for the Boys, Guidelines for the Girls: A Qualitative Study of the Factors Influencing Gender Differences in Symptom Reporting During Childhood and Adolescence.

[b13] MacLean A, Sweeting H, Hunt K (2010). ‘Rules’ for boys, ‘guidelines’ for girls: gender differences in symptom reporting during childhood and adolescence. Social Science & Medicine.

[b14] Michell L, Kitzinger J, Barbour RS (1999). Combining focus groups and interviews: telling how it is, telling how it feels. Developing Focus Group Research.

[b15] Morgan DL (1997). Focus Groups as Qualitative Research.

[b16] Moses T (2010). Being treated differently: stigma experiences with family, peers, and school staff among adolescents with mental health disorders. Social Science & Medicine.

[b17] Potts Y, Gillies ML, Wood SF (2001). Lack of mental well-being in 15-year-olds: an undisclosed iceberg?. Family Practice.

[b18] Punch S (2002). Interviewing strategies with young people: the ‘secret box’, stimulus material and task-based activities. Children & Society.

[b19] Rickwood D, Braithwaite V (1994). Social-psychological factors affecting help-seeking for emotional problems. Social Science & Medicine.

[b20] Ritchie J, Lewis J (2003). Qualitative Research Practice. A Guide for Social Science Students and Researchers.

[b21] Roose GA, John AM (2003). A focus group investigation into young children’s understanding of mental health and their views on appropriate services for their age-group. Child: Care Health & Development.

[b22] Rutter M, Smith DJ (1995). Psychosocial Disorders in Young People: Time Trends and Their Causes.

[b23] Santor DA, Poulin C, LeBlanc JC, Kusumakar V (2007). Facilitating help seeking behaviour and referrals for mental health difficulties in school aged boys and girls: a school-based intervention. Journal of Youth & Adolescence.

[b24] Secker J, Armstrong C, Hill M (1999). Young people’s understanding of mental illness. Health Education Research.

[b25] Spratt J, Shucksmith J, Philip K, Watson C (2010). ‘The bad people go and speak to her’: young people’s choice and agency when accessing mental health support in school. Children & Society.

[b26] Sweeting H, West P (2003). Sex differences in health at ages 11, 13 and 15. Social Science & Medicine.

[b27] Sweeting H, West P, Young R, Der G (2010). Can we explain increases in young people’s psychological distress over time?. Social Science & Medicine.

[b28] Timlin-Scalera RM, Ponterotto JG, Blumberg FC, Jackson MA (2003). A grounded theory study of help-seeking behaviours among white males high school students. Journal of Counselling Psychology.

[b29] Torsheim T, Ravens-Sieberer U, Hetland J, Valimaa R, Danielson M, Overpeck M (2006). Cross-national variation of gender differences in adolescent subjective health in Europe and North America. Social Science & Medicine.

[b30] Wade T, Carney J, Pevalin D (2002). Emergence of gender differences in depression during adolescence: national panel results from three countries. Journal of the American Academy of Child and Adolescent Psychiatry.

[b31] Williams C (2000). Doing health, doing gender: teenagers, diabetes and asthma. Social Science & Medicine.

[b32] Williams B, Pow J (2007). Gender differences and mental health: an exploratory study of knowledge and attitudes to mental health among Scottish teenagers. Child and Adolescent Mental Health.

